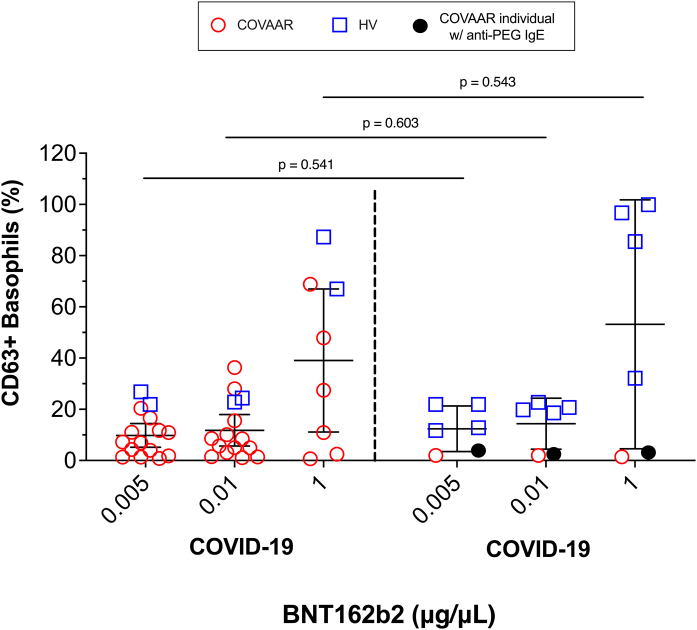# Basophil activation test to BNT162b2 lacks specificity for predicting allergic reactions to the mRNA vaccine

**DOI:** 10.1016/j.jacig.2025.100495

**Published:** 2025-05-13

**Authors:** Sydney Ann Kee, Ana Olivera, Lindsay Chatman, Muhammad B. Khalid, Min Jenny Li, Eric Chu, Ellen Zektser, Karen Laky, Pamela A. Frischmeyer-Guerrerio

**Affiliations:** a Laboratory of Allergic Diseases, National Institute of Allergy and Infectious Diseases, National Institute of Health, Bethesda, Md; b Clinical Monitoring Research Program Directorate, Frederick National Laboratory for Cancer Research, Frederick, Md

**Keywords:** Basophil activation test, BAT, COVID-19 mRNA vaccine, vaccine reactions, vaccine allergy, polyethylene glycol, PEG

## Abstract

**Background:**

Allergic reactions to the coronavirus 2019 disease (COVID-19) mRNA vaccine (BNT162b2) were originally reported at higher rates than expected, contributing to vaccine hesitancy and, in some cases, unnecessary vaccine avoidance. Identification of a test that accurately predicts allergic reactions to mRNA vaccines is critical to improve patient care, particularly given the growing use of mRNA-based technologies.

**Objective:**

We sought to determine the value of basophil activation tests (BATs) in predicting allergic reactions to the BNT162b2 vaccine.

**Methods:**

Blood from 16 participants enrolled in the clinical trial COVID Vaccine Allergy Reaction (COVAAR [ClinicalTrials.gov identifier NCT04977479]) who reported a systemic allergic reaction to their first dose of the COVID-19 mRNA vaccine was drawn before the second or booster dose and incubated with varying concentrations of the BNT162b2 vaccine or the vaccine component dimyristoyl glycerol–polyethylene glycol 2000. Basophil activation was quantified by CD63 expression via flow cytometry. In addition, 8 healthy volunteers (HVs) who tolerated the vaccine were included as controls.

**Results:**

Basophil responses to dimyristoyl glycerol–polyethylene glycol 2000 or the BNT162b2 vaccine were not higher among the COVAAR participants than among the HVs. Basophil responses did not correlate with time elapsed since last vaccine administration or previous COVID-19 infection. Instead, in both the HV and COVAAR groups, basophil reactivity was greater among those individuals who had received 2 or more vaccine doses than in those who had received only 1 dose.

**Conclusion:**

The BAT cannot predict allergic reactions to the BNT162b2 vaccine, and number of previous vaccinations received could be a confounding factor for interpreting the results of the BAT. Further studies are necessary to find a test that can accurately predict allergic reactions to the mRNA vaccine.

## Introduction

Although rare, allergic reactions caused by vaccination are a serious concern for individuals with allergy, and they contribute to vaccine hesitancy. This issue became heightened during the coronavirus disease 2019 (COVID-19) pandemic, when the incidence of allergic reactions to mRNA COVID-19 vaccines was initially reported to be higher than the incidence of reactions to traditional vaccines. Given the benefits of vaccines in public health, the development of tests that reliably predict allergic reactions is crucial for safe patient management and vaccination completion. One impediment to evaluating these tests has been the low incidence of vaccine-induced allergic reactions. Mass vaccinations during the COVID-19 pandemic provided an opportunity to identify sizable cohorts of individuals with allergy and evaluate the tests commonly used in allergologic workups as potential predictive tools of allergic reactions to the vaccine. Understanding the data collected from these tests is crucial for the future of medicine, as the liposome-based delivery of mRNA is a model platform that will likely be used for many vaccines and therapeutics.

Basophils and mast cells are key initiators of allergic reactions. Diagnostic allergy tests include skin prick tests (SPTs) and basophil activation tests (BATs) to measure mast cell or basophil degranulation, respectively, in response to suspected allergens. mRNA COVID-19 vaccines and their excipients have been evaluated in both SPTs and BATs to predict hypersensitivity reactions to the vaccine. mRNA COVID-19 vaccines deliver viral protein-encoding mRNA in nanoparticles coated with lipid-conjugated polyethylene glycol (PEG) 2000 (PEG 2000) on the surface. Because PEG is found in numerous personal care products and medications, it was initially implicated as a culprit for vaccine reactions in sensitized individuals.^1^ Although SPTs are noninvasive and provide rapid results, several studies using vaccine and PEG 2000 found SPTs to be unreliable predictors of an allergic reaction to the mRNA vaccine.^1^ BATs have also been tested in cohorts of mRNA vaccine–reactive individuals, with promising results in predicting hypersensitivities.^2^, ^3^, ^4^ However, reports on BATs have noted that using PEG conjugated to lipid nanoparticles (dimyristoyl glycerol–PEG [DMG-PEG]) better predicts PEG reactions than “naked” unconjugated PEG^5^ does and that prior COVID-19 infections can increase basophil reactions to the vaccine.^6^

## Results and discussion

We sought to evaluate the predictive value of BATs for vaccine reactions in a cohort enrolled in the clinical trial COVID Vaccine Allergy Reaction (COVAAR [ClinicalTrials.gov identifier NCT04977479]), which was conducted to determine whether individuals who reported systemic allergic reactions to their first dose of the mRNA vaccine could safely receive subsequent doses. A total of 16 consenting participants from COVAAR were enrolled to receive their second and booster doses of the COVID-19 mRNA vaccine (BNT162b2) under observation.^7^ Although all of the participants reported convincing systemic allergic reactions to their first vaccine dose, only 2 individuals met anaphylaxis criteria after both their second and booster doses. Immunization stress–related responses (ISRRs), caused by the anxiety associated with the vaccination process rather than an immune response to a vaccine component, were common.^7^

BATs were performed before administration of the second (n = 13) or booster dose (n = 3). A total of 8 age- and sex-matched healthy volunteers (HVs) with no reaction to the vaccine were recruited as controls. For the BATs, blood samples were incubated with DMG-PEG 2000 or the BNT162b2 vaccine for 20 minutes at 37^°^C. Basophil (SSC^lo^CD123^+^HLA-DR^–^) activation was quantified by measuring expression of CD63, an activation marker, on the cell surface by flow cytometry. Saline was used as a negative control, and stimulation with anti-IgE used as a positive control to identify nonresponders.^8^ In all, 3 nonresponders were identified and excluded from the analyses. Notably, this included 1 of the 2 COVAAR participants, who experienced bona fide allergic reactions to all 3 vaccinations.^7^ The other COVAAR participant with repeated systemic allergic reactions who was included in the BAT analyses, experienced throat itching, repetitive coughing and chest tightness, generalized itching and flushing, and periorbital swelling after the second vaccine dose, as well as cough, throat tightness and itchiness, facial flushing, and hypotension responsive to epinephrine after the booster dose. Comparisons between groups were made by using unpaired 2-sample 2-sided Welch *t* tests to determine statistical significance (*P* < .05).

**Fig 1:**
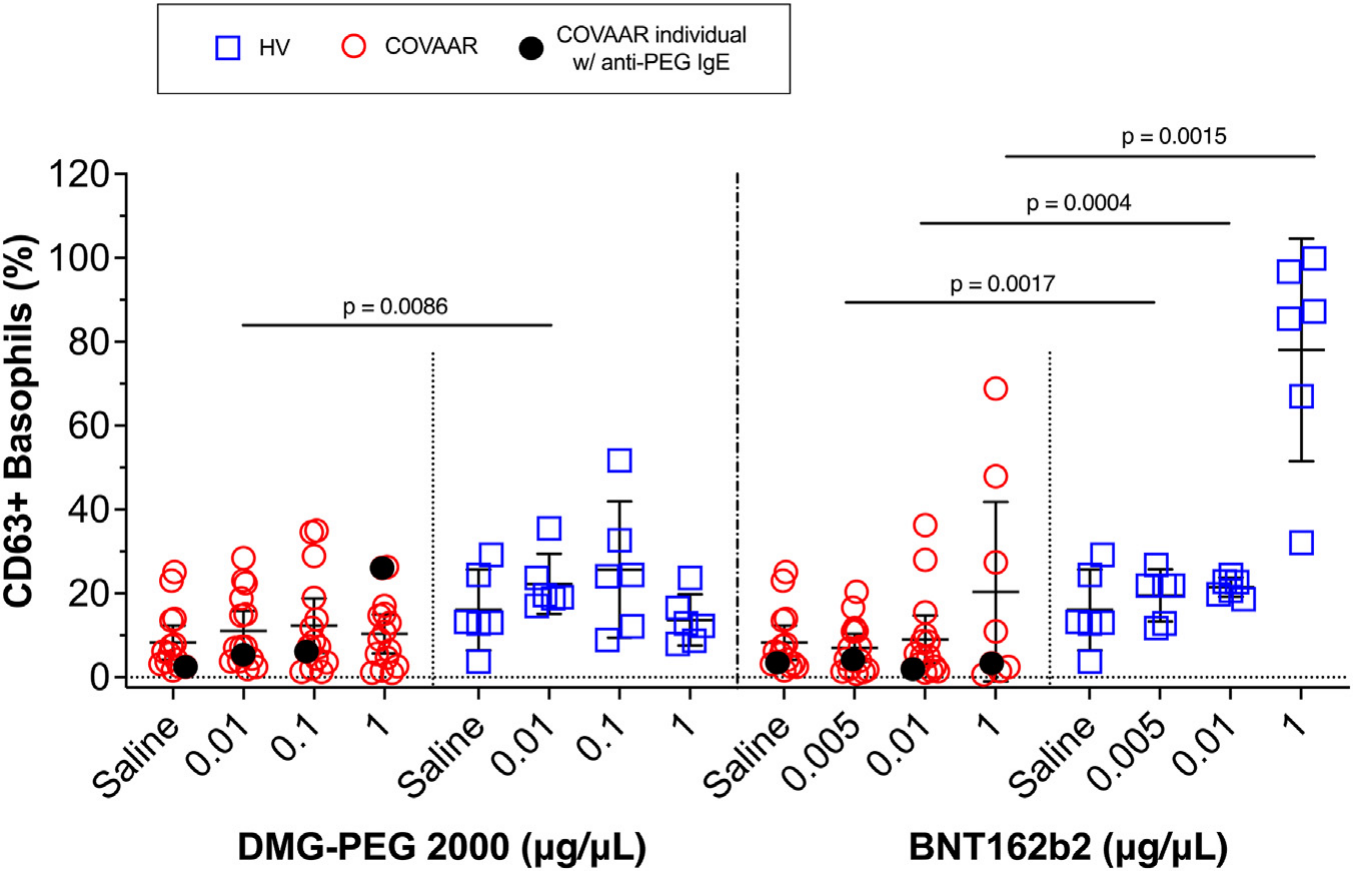
Basophil reactivity to DMG-PEG 2000 or the BNT162b2 vaccine is comparable between HVs and COVAAR individuals. Percentage of basophils expressing CD63 in response to PEG-2000 or the BNT162b2 vaccine. PEG-2000 concentrations were in the range of those in the vaccine (0.05 mg of PEG-2000/dose). Not all concentrations of the BNT162b2 vaccine were tested in every COVAAR participant. Data represent means with 95% CIs and *P* values from univariant *t* tests.

**Supplementary Fig E1:**
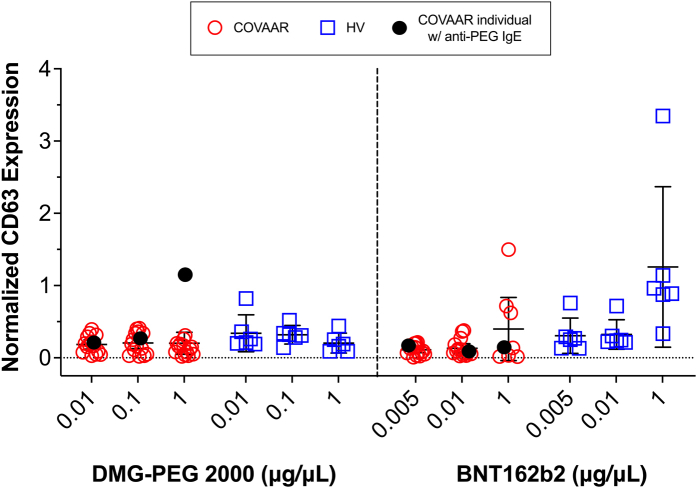


Some reports have suggested that a positive BAT result to PEG may predict vaccine reactions,^3^^,^^6^^,^^9^ but the underlying causes remain unclear. Both classic IgE-dependent mechanisms (which usually lead to recurrent allergic responses on subsequent exposure) and pseudoallergic, IgE-independent mechanisms (which may not be associated with recurrent reactions) have been proposed. However, in our study, basophil activation in response to DMG-PEG 2000 was largely comparable in the COVAAR participants and the HVs (Fig 1). This included the COVAAR participant with confirmed anti-PEG IgE and anti-PEG IgG antibodies and repeated allergic reactions to the mRNA vaccine (filled circle in Fig E1 in the Online Repository at www.jaci-global.org).^1^ This individual showed the highest reactivity to a dose of 1 μg/μL of DMG-PEG 2000 only when the basophil responses to DMG-PEG 2000 were normalized to their IgE responses (see Fig E1). Furthermore, the basophils from the COVAAR group, including this individual, did not show higher responses to the BNT162b2 vaccine (which also presents PEG on the nanoparticle surface) than the HVs did (Fig 1 and see Fig E1).

**Fig 2:**
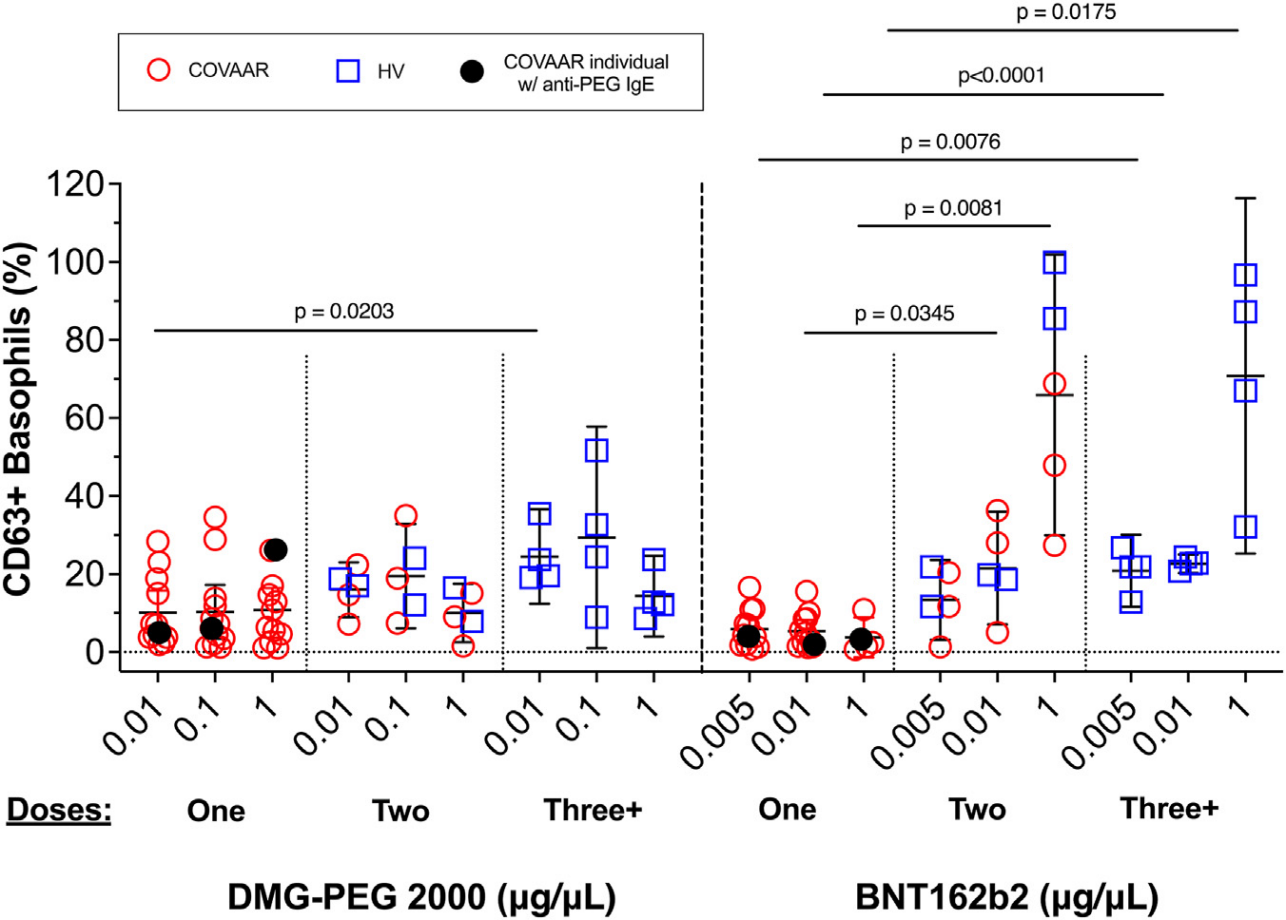
Basophil reactivity to the BNT162b2 vaccine, but not to PEG-2000, increased with the number of vaccinations in both the HVs and the COVAAR individuals. Percentage of basophils expressing CD63 in response to DMG-PEG 2000 or the BNT162b2 vaccine. All participants were grouped by number of vaccinations received. No statistical differences between the HVs and COVAAR subjects were found in the 2-dose subset when a univariant *t* test was used. The BNT162b2 vaccine at a dose of 1 μg/μL was tested in all HVs and in 8 COVAAR individuals (5 who had received 1 dose of the vaccine and 3 participants who received 2 doses) because of the shortage of vaccine availability at the time of the visits. The BNT162b2 vaccine at a dose of 0.01 μg/μL was tested in all participants.

Surprisingly, we observed significantly higher basophil responses in the HV group than in the COVAAR group at all doses of the BNT162b2 vaccine tested (Fig 1). This contrasts with the findings of previous studies showing higher basophil responses to the BNT162b2 vaccine in vaccine-reactive individuals than in HVs.^2^^,^^4^^,^^5^ We considered a few explanations to account for this discrepancy. One consideration was the time elapsed between the BAT assay and last vaccination; however, no correlation was found (*R*^*2*^ = 0.04243; *P* = .5949). Nor could prior COVID-19 infection explain the basophil responses (see Fig E2 in the Online Repository at www.jaci-global.org), contrasting with the findings of another report.^6^ We then hypothesized that basophil reactivity to the BNT162b2 vaccine may reflect the number of vaccinations received. In our cohort, the HVs were fully vaccinated and boosted, whereas the COVAAR participants avoided further vaccinations after their first dose. When combining data from the HVs and COVAAR subjects and segregating by the number of vaccinations, we observed significantly higher average basophil responses to the BNT162b2 vaccine in those individuals who had received more than 1 vaccination, whereas the responses to DMG-PEG 2000 were largely unaffected (Fig 2). Thus, number of vaccinations could potentially explain the range of basophil responses to the BNT162b2 vaccine among vaccine-reactive and vaccine-tolerant individuals that were reported across studies. It is likely that in studies performed early in the pandemic (before vaccines were readily available), HVs were unvaccinated or had only received 1 dose.

We conclude that the results of BATs in response to the BNT162b2 vaccine are not reliable predictors of an allergic reaction to the vaccine. First, we noted that receiving more than 1 dose of the vaccine was associated with higher basophil reactivity to the BNT162b2 vaccine (Fig 2). Second, the BAT to the BNT162b2 vaccine failed to identify a participant with anti-PEG IgE and anti-PEG IgG who experienced repeated systemic allergic reactions in response to vaccination. Lastly, the results of BATs are not interpretable in 10% to 20% of the population because those individuals are nonresponders.^8^

Although the limited size of our cohort did not allow for multivariate analyses, our data suggest that the number of vaccinations received could be a confounding factor for interpreting BAT results. This is crucial because the delivery of lipid-based nanoparticles will likely extend to other vaccines and medications, and repeated exposures would make BATs unreliable for predicting reactions to such therapeutics. Positive results of BATs to the BNT162b2 vaccine have been attributed to IgE-dependent^4^, ^5^, ^6^ and IgE-independent^9^ mechanisms in individuals with vaccine hypersensitivities, but the mechanism in healthy individuals requires further investigation. The search for a test that accurately predicts an allergic reaction to vaccination remains a challenge, highlighting the importance of understanding the underlying causes for BAT reactivity.

## Disclosure statement

Supported by the Division of Intramural Research of the 10.13039/100000060National Institute of Allergy and Infectious Diseases and, in part, with federal funds from the 10.13039/100000054National Cancer Institute of the 10.13039/100000002National Institutes of Health (contract HHSN261201500003I or 75N91019D00024). The content of this publication does not necessarily reflect the views or policies of the Department of Health and Human Services, nor does mention of trade names, commercial products, or organizations imply endorsement by the US Government.

Disclosure of potential conflict of interest: L. Chatman is employed by the Kelly contracting agency at the National Institute of Allergy and Infectious Diseases of the National Institutes of Health. The rest of the authors declare that they have no relevant conflicts of interests.

## Figures and Tables

**Supplementary Fig E2:**