# Supplementary material for: Basophil activation test to BNT162b2 lacks specificity for predicting allergic reactions to the mRNA vaccine
Source: J Allergy Clin Immunol Glob. 2025 May 13;4(3):100495. doi: 10.1016/j.jacig.2025.100495 (PMC12151657; doi:10.1016/j.jacig.2025.100495)
Supplement: Supplementary Data [file mmc1.docx]

**Figure E1: Normalized basophil CD63 expression highlights a COVAAR participant with anti-PEG IgE antibodies as reactive to DMG-PEG 2000 but not BNT162b2.** Basophil reactivity to the indicated concentrations of DMG-PEG 2000 or BNT162b2 was normalized in each participant to their maximal basophil response to anti-IgE ($\frac{\% CD63+ to PEG or BNT162b2}{\% CD63+ to maximal anti-IgE}$ ). The filled circle denotes the COVAAR participant with anti-PEG IgE antibodies.

**Figure E2: Prior COVID-19 infection is not a determining factor of basophil reactivity to BNT162b2.** Basophil reactivity to the indicated concentrations of BNT162b2 in COVAAR participants and healthy volunteers (HV) grouped by COVID infection history. Average responses from uninfected individuals (COVAAR n=13, HV n=2) and previously infected (COVAAR n=2, HV n=4) were not statistically significant using an unpaired t-test.
